# Ceria/polymer nanocontainers for high-performance encapsulation of fluorophores

**DOI:** 10.3762/bjnano.10.53

**Published:** 2019-02-22

**Authors:** Kartheek Katta, Dmitry Busko, Yuri Avlasevich, Katharina Landfester, Stanislav Baluschev, Rafael Muñoz-Espí

**Affiliations:** 1 Max Planck Institute for Polymer Research, Ackermannweg 10, 55128 Mainz, Germany; 2 Optics and Spectroscopy Department, Faculty of Physics, Sofia University “St. Kliment Ochridski” 5 James Bourchier, 1164 Sofia, Bulgaria; 3 Institute of Materials Science (ICMUV), Universitat de València, c/ Catedràtic José Beltrán 2, 46980 Paterna, Spain

**Keywords:** cerium oxide, crystallization, miniemulsion, nanocapsule, photoluminescence, singlet oxygen

## Abstract

We report the synthesis of high-performance organic–inorganic hybrid fluorescent nanocapsules comprising a polymer shell armored with an inorganic layer and a liquid core containing a fluorophore. The polymeric capsules are synthesized by free radical miniemulsion polymerization and contain covalently bound carboxylate surface functionalities that allow for the binding of metal ions through electrostatic interaction. A cerium(IV) oxide nanoparticle layer, formed in situ at the surface of the hybrid nanocapsules, acts as oxygen scavenger and keeps external reactive molecular oxygen from entering into the capsules, eventually resulting in a reduction of the photooxidation of encapsulated fluorescent molecules. This approach shows an increase in the fluorescence of the model organic fluorophore terrylene diimide by avoiding the ground-state molecular oxygen to react with electronically excited states of the fluorescent hydrocarbon molecule.

## Introduction

Hybrid polymeric core–shell nanoparticles with encapsulated fluorescent dye molecules are frequently employed for life-science applications, such as cell labeling and drug delivery [1–5]. In recent years, polymeric hybrid nanoparticles and nanocapsules with tailored inorganic components on the surface have attracted great interest because of the possibility to tune size, composition, porosity, stability, surface functionality, and colloidal stability [6–12]. Generally, fluorescent dye molecules are sensitive to the external environment, which leads to unwanted chemical reactions [13]. Molecular oxygen is a well-known quencher of fluorescence. Consequently, the shell properties of the nanocontainers are often customized to protect the fluorophore encapsulated into a liquid core.

Higher lying singlet states of a fluorophore are relatively short living and quickly relax to the lowest excited singlet state, relaxing further to the excited triplet state via an intersystem crossing process. The ground state of the oxygen molecule is a triplet state and the energy of the excited triplet state of the fluorophore can be transferred to the oxygen molecule. Thus, singlet oxygen is obtained through the process of fluorescence quenching [13–16].

Photooxidation significantly changes the emission properties of fluorophores because of an irreversible chemical reaction. In a simple description, the longer the fluorescence lifetime and the lower the fluorescence quantum yield of the fluorophore are, the stronger is the sensitivity to the presence of oxygen quenching [13]. The solubility of oxygen in organic solvents is also an important factor in the quenching process of a florescent molecule. The solubility of oxygen in organic solvents decreases with increasing alkyl chain length of the alkanes [17–18]. Furthermore, the oxygen quenching is less efficient in high-viscosity media [13]. In bulk fluorescent samples with a dye content between 10^−4^ and 10^−5^ M, oxygen quenching does not play a significant role. By applying standard degassing techniques such as nitrogen or argon bubbling and/or freeze–pump–thaw cycles, the oxygen content can be lowered. These techniques are efficient enough to suppress the oxygen quenching of fluorescence [13]. The case of fluorescent samples containing dyes encapsulated in nanoconfined materials is more complicated. In general, the encapsulation process of the dye leads to a drastic reduction of the total dye concentration in the sample and increases the amounts of dissolved molecular oxygen and encapsulated fluorescent dye.

A variety of methods have been applied to encapsulate the fluorescent materials into micro- and nanocapsules [19–20], with a concentration of hydrophobic dye of the order of 10^−4^ M and a total solid content of 1–5 wt %. In bulk fluorescent samples, the oxygen/dye molar concentration ratio is typically about 10:1, while for nanoconfined materials is of about 200:1. This huge excess of oxygen affects the optical response of nanometric systems not only by quenching dramatically the fluorescence but also by photooxidation of the organic dye [21]. This effect is an additional source of uncertainty for quantitative measurements in life sciences [22–23].

Near-infrared (NIR) light undergoes less diffuse scattering than visible light, causes less photodamage, and can penetrate deeper into tissues. Hence, it is preferred for life-science applications. Organic dyes that can be excited above 600 nm are highly favorable for live-cell imaging experiments, because the background signal obtained from the autofluorescence of living cells is negligible in the near-infrared region [24–25]. In this work, we have chosen a specific fluorescent dye molecule, i.e., terrylene diimide (TDI), which shows intense fluorescence in the NIR spectral region of the electromagnetic spectrum, to be encapsulated in polymeric nanocapsules [26–27]. TDI, which than can be excited with red light (λ_exc_ = 633 nm from a HeNe laser, for instance) and shows bright fluorescence at λ_max_ = 670–690 nm, is a suitable organic dye for patterning or imaging of biomaterials. This dye belongs to the rylene family [28], which is formed by extending the π-conjugated core system of highly fluorescent perylene-3,4:9,10-tetracarboxdimides. The rylene dyes have unique optical properties, such as high extinction coefficients, high thermal, chemical and photochemical stabilities, and exhibit brilliant colors [29–30]. Furthermore, in bulk samples, TDI demonstrates an excellent fluorescence quantum yield (ca. 90%) [24], less affected by the presence of molecular oxygen. Using the example of TDI, we aim to demonstrate that in nanoconfined geometries, quenching of the fluorescence of a dye becomes an important issue that needs to be taken into account. To obtain sustainable and reproducible results, the efficient protection against oxygen quenching cannot be neglected because the fluorescent response is used as a quantitative measure. Here, we focus on the development of an oxygen-protection strategy that is applicable for a broad range of encapsulated materials.

The core–shell structure of the hybrid organic–inorganic nanoparticles allows for the independent molecular design of each part. For instance, the oxygen permeability of the shell material can be lowered drastically by using semicrystalline nanocellulose [31]. Furthermore, a bovine serum albumin film at the oil–water interface [32] or rose bengal embedded in a microcapsule shell [33] can also be used to protect the encapsulated dye from molecular oxygen. Incorporating oxygen-scavenging materials such as WO_3_ photocatalysts loaded with Pt [34], phosphonate coatings [35], organophosphates [36] or polyoxyethanyl α-tocopheryl sebacate [37] significantly reduces oxygen quenching. Last but not least, another possibility is to attach an oxygen scavenger (e.g., diphenylanthracene moieties) to the emissive dye itself [38]. Decoration of the molecular structure of cyanine dyes including a cyclodextrin complex, acetyl modification, fluoro- and cyano-substitution leads to increased photostability of the fluorophore [5]. However, unfortunately, all these oxygen-protection strategies affect the properties of core and shell materials that are used to form nanoconfined materials.

Our main goal is to develop a strategy to reduce the impact of reactive oxygen by applying an additional synthetic step, while keeping unchanged all parameters involved in the synthesis of the initial nanoconfined materials. Exemplarily, polystyrene nanocontainers with a liquid hydrophobic core of hexadecane synthesized by using miniemulsion techniques will be protected by depositing metal-oxide particles on the surface, as proposed in a previous minireview from our team [21]. Deposition of metal-oxide particles on the surface of polymer hybrid nanoparticles via controlled surface crystallization was shown in previous works of our group [10,12]. The regular arrangement of functional groups on the nanocapsule surface can provide nucleation and structure-directing centers for the controlled crystallization of metal-oxide particles. We have chosen cerium(IV) oxide nanoparticles to be deposited on the nanocapsule surface in order to combine biocompatibility with a high oxygen-scavenging ability. Cerium oxide is a lanthanide metal oxide with a redox potential behavior that can easily switch between cerium(IV) and cerium(III) and has the capability to leave oxygen vacancies in the crystal lattice [39]. Cerium(IV) oxide exhibits excellent antioxidant properties, ideal for applications such as water-gas shift catalysis [40], combustion catalysis [41], oxygen ion conductors, and solid-oxide fuel cells [42]. Due to the valence and oxygen defect properties of cerium(IV) oxide, nanoparticles of this material are also used as efficient free-radical scavengers in biomedical applications as a potent therapeutic option for the treatment of disorders generated by reactive oxygen species, such as neurodegenerative disorders, retinal disorders and cancer [43–45].

In this work, we report the process of armoring anionically functionalized nanocontainers loaded with TDI by crystallizing cerium(IV) oxide nanoparticles on the nanocontainer surface. As a result, we obtain reproducible and sustained fluorescence of the nanocapsules, independently from the oxygen content of the external environment.

## Experimental

### Materials

Styrene (S, ≥99.0%) acrylic acid (AA, Sigma-Aldrich, 99%), hexadecane (Sigma-Aldrich, 99.0%), tetrahydrofuran (THF, Sigma-Aldrich, ≥99.9%), cerium(III) nitrate hexahydrate (Sigma-Aldrich, 99.99%), sodium hydroxide (Sigma-Aldrich, ≥97.0%), ammonia solution (28% aqueous solution, VWR), sodium dodecyl sulfate (SDS, Alfa Aesar, 99%), and 2,2'-azobis(2-methylbutyronitrile) (V59, Wako) were used as received. Styrene was passed over an aluminium oxide column to remove the stabilizer before use.

### Synthesis of the dye and preparation of its stock solution

*N*,*N*’-(2,6-Diisopropylphenyl)-1,6,9,13-tetrakis[4-(1,1,3,3-tetramethylbutyl)phenoxy]terrylene-3,4,11,12-bis(dicarboximide) (TDI) was synthesized according to [26] (molecular structure and absorption/fluorescence emission spectra are given in Supporting Information File 1, Figures S1 and S2, respectively). The prepared TDI was dissolved in sufficient amounts of THF and transferred in hexadecane (4 g) to obtain a final concentration of 1 × 10^−4^ M. Afterwards, THF was completely removed by rotary evaporation (1 h at 70 mbar and 50 °C).

### Synthesis of carboxyl-functionalized nanocapsules

Polystyrene nanocapsules were synthesized either under ambient conditions (sample NC) or under argon atmosphere [sample NC(Ar)] by free-radical miniemulsion polymerization [46]. The continuous phase contains SDS (30 mg) and demineralized water (30 g). The disperse phase contains styrene (1.8 g) and acrylic acid (0.2 g) as monomers, hexadecane (4 g) with the fluorescent dye TDI and the initiator V59 (100 mg). The two phases were mixed and pre-emulsified by stirring at 1000 rpm for 1 h. The emulsion was prepared by ultrasonication (Branson Digital Sonifier 450-D; 1/2″ tip, 90% intensity, 2 min) while cooling in an ice-water bath to avoid polymerization due to heating. The polymerization reaction was carried out in a closed flask at 72 °C for 18 h under constant stirring. The capsule content (polymer + hexadecane), determined gravimetrically by weighing 0.500 g of suspension before and after freeze-drying, was of ca. 15 wt %. The term “capsule content” is used as an analogous to the term “solid content” for solid capsules to take into account the fact that the hexadecane core is also weighted and counted. Since hexadecane is not solid under the conditions of measurement, the term “solid contend” would not be appropriate.

Synthesized nanocapsules were further used for the cerium-oxide crystallization experiments with specific concentration. The surface charge density was determined at pH 10 to ensure the complete deprotonation of the carboxylic groups.

### Synthesis of CeO_2_/polymer hybrid nanocapsules

The synthesized polymeric nanocapsules, NC and NC(Ar), were used in the crystallization experiments to obtain the hybrid samples, NC-CeO_2_ and NC(Ar)-CeO_2_, respectively. We apply an analogous procedure to the one used for solid particles [10,12]. The experiment was carried out at 35 °C in a closed flask under constant stirring while keeping the pH value constant during the whole procedure. First, the pH value of the dispersion was adjusted to pH 10 with a 28% ammonia solution. Then 5 mmol of metal salt per gram of dispersion nanocapsules was added and the dispersion was stirred for 2 h to allow for the binding of cerium ions to the surface of the capsules. Cerium(III) nitrate hexahydrate was dissolved in 2 mL of demineralized water and 200 µL of the latex dispersion was added to the solution. Afterwards, the precipitation of the oxide was carried out by adding dropwise 2 mL of aqueous NaOH solution (0.1 M) using a syringe pump (dropping speed of 1 mL·h^−1^). The addition of the base leads to the precipitation of Ce(OH)_3_, which is insoluble in water. In an alkaline environment, Ce(OH)_3_ is spontaneously oxidized to hydrated Ce(IV) ions by the oxygen of the environment, and then hydrolyzed to form hydroxocomplexes, which eventually evolve to CeO_2_ [47]. The mixture was further stirred for 24 h to complete oxide formation. The loaded samples were freeze-dried for subsequent characterizations.

### Characterization of the materials

Particle sizes of the prepared nanocapsules were determined by dynamic light scattering (DLS) using a Nicomp 380 PSS particle sizer (Santa Barbara, CA) at a fixed angle of 90°. Table 1 reports the different samples reported in this work with the corresponding particle sizes measured by DLS.

**Table 1:** Characteristics of the samples reported in this work.

sample	material	atmosphere during synthesis	diameter (nm)^a^

NC	poly(styrene/acrylic acid)	air	140 (±23%)
NC(Ar)	poly(styrene/acrylic acid)	argon	153 (±29%)
NC-CeO_2_	poly(styrene/acrylic acid) + CeO_2_	air	146 (±30%)
NC(Ar)-CeO_2_	poly(styrene/acrylic acid) + CeO_2_	argon	159 (±18%)

^a^Determined by DLS.

The surface charge density of the negatively charged latex nanocapsules was determined by direct polyelectrolyte titration with a roughly 0.001 N solution of poly(diallyldimethyl ammonium chloride), detecting the end point with an automatic streaming current detector with a particle-charge detector Mütek PCD-03 in combination with a Metrohm Titrino automatic titrator. The samples were diluted to a solid content of 0.1 wt % for titration and the surface charge density of 1.6 carboxylic groups per nm^2^ was obtained for samples NC and NC(Ar).

Transmission electron microscopy (TEM) was carried out with a Jeol 1400 microscope at an acceleration voltage of 200 kV. Samples for TEM observation were prepared by dropping the diluted dispersions on carbon-coated copper grids and dried. Scanning electron microscopy (SEM) with a LEO Gemini 1530 field-emission microscope operated with an extractor voltage of 0.7 kV. Samples for SEM observation were prepared by dropping the diluted dispersions on small silicon wafers that were subsequently dried. EDX analysis combined with elemental mapping was carried out in a Hitachi SU8000 SEM microscope equipped with a Bruker AXS spectrometer with an operation voltage of 5 kV.

X-ray diffraction of the freeze dried sample was conducted on a Philips PW 1820 diffractometer with monochromatic Cu Kα radiation (λ = 1.5418 Å, 40 kV, 30 mA, 5 s, Δθ = 0.02). Thermogravimetric analysis of the freeze-dried sample was conducted using a Mettler Toledo TGA-851 at a heating rate of 10 °C·min^−1^.

Fluorescence spectra were recorded for the dispersions containing 0.75 wt % capsule content by using in a custom-built setup containing a diode laser with wavelength 635 nm (Roithner Lasertechnik GmbH, ADL-63201TL) with an excitation intensity of 0.2 W·cm^−2^. It is important to note that all fluorescence emission measurements were carried out by using the same batch of nanocapsules at the same capsule content. A first batch of nanocapsules was prepared under ambient conditions and an analogous one in argon atmosphere.

## Results and Discussion

The objective of this work was to design colloidally stable polystyrene-based hybrid nanocapsules containing the fluorescent dye terrylene diimide (TDI) and being armored with metal-oxide nanoparticles on the polymer shell surface. Polymeric nanocapsules (labeled as sample NC) were prepared under ambient conditions by free radical miniemulsion polymerization of styrene and acrylic acid. The thickness of the shell of the formed nanocapsules was ca. 20 nm in average, and the weight ratio polymer/hexadecane was ca. 1:2. The fluorescent dye was dissolved in the liquid hexadecane core before polymerization of the shell. The nanocapsule formation takes place by phase separation between the formed polymer and hexadecane [46]. The surface of the nanocapsules was negatively charged, as proven by polyelectrolyte titration (surface charge density of 1.6 nm^−2^ at pH 10), which is a result of the used anionic surfactant (sodium dodecyl sulfate) and the hydrophilic co-monomer (acrylic acid). Acrylic acid plays a crucial role in binding the cerium ions to the surface of nanocapsules and is also helpful to increase the hydrophilic nature of the system.

**Figure 1:**
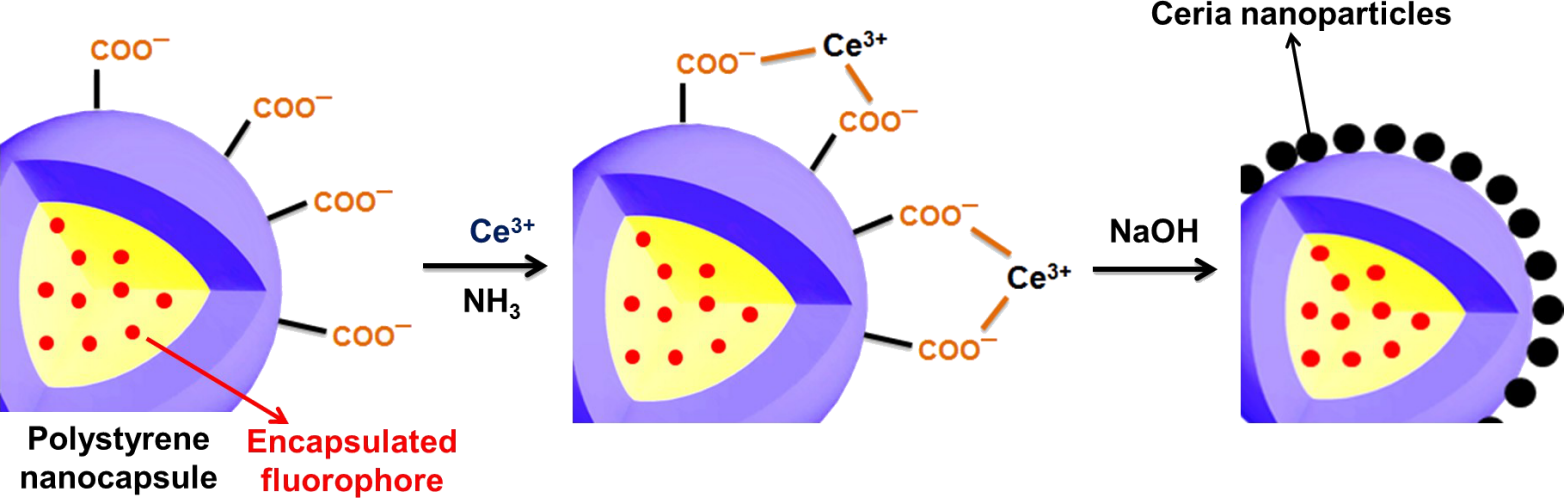
Schematic representation of the formation of cerium(IV) oxide on the surface of carboxylate functionalized fluorescent nanocapsules.

**Figure 2:**
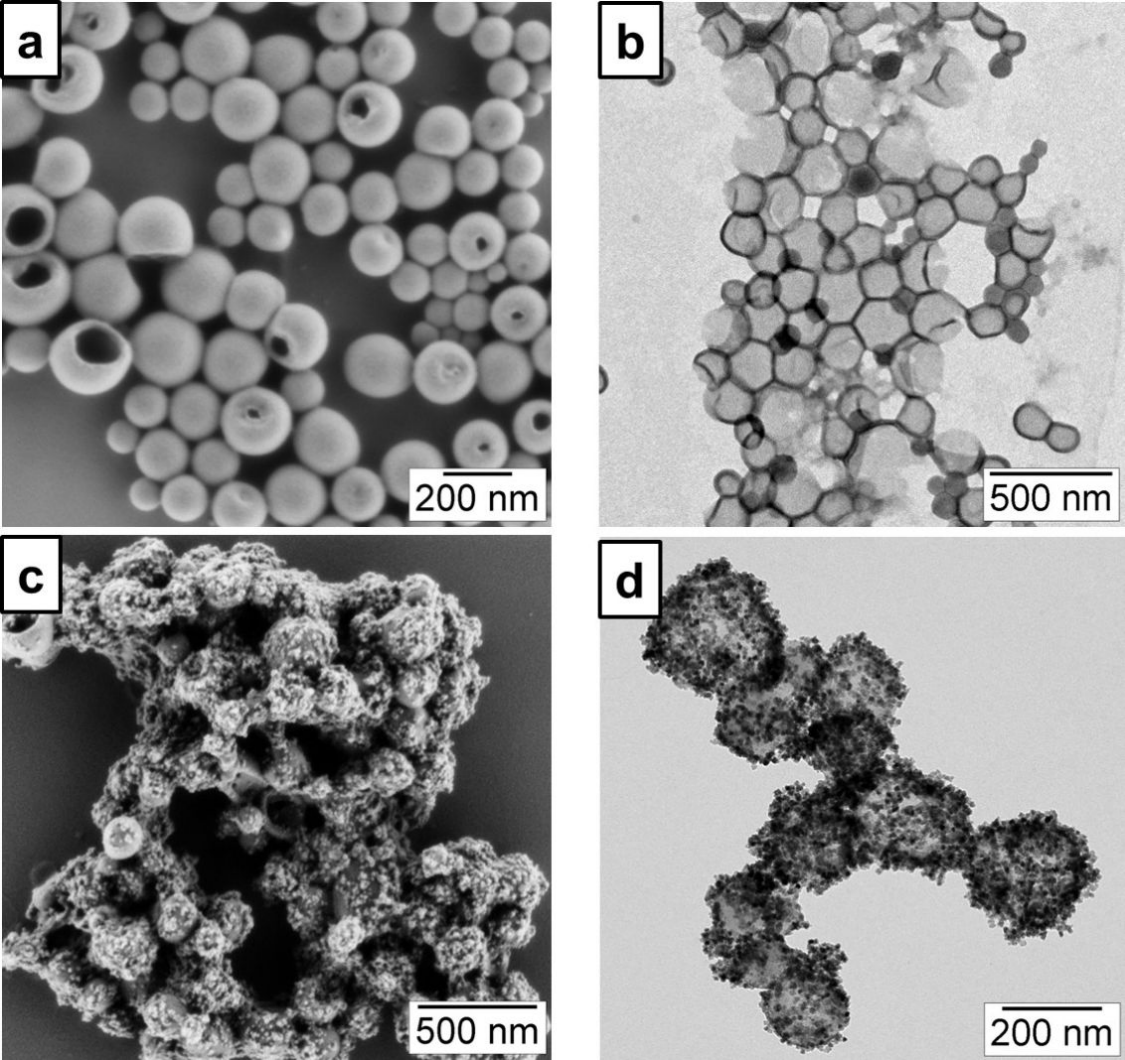
Electron micrographs of the prepared capsules: a) SEM and b) TEM of carboxylate-functionalized polystyrene hybrid nanocapsules (sample NC); c) SEM and d) TEM of ceria/polymer hybrid samples (sample NC-CeO_2_) [21].

**Figure 3:**
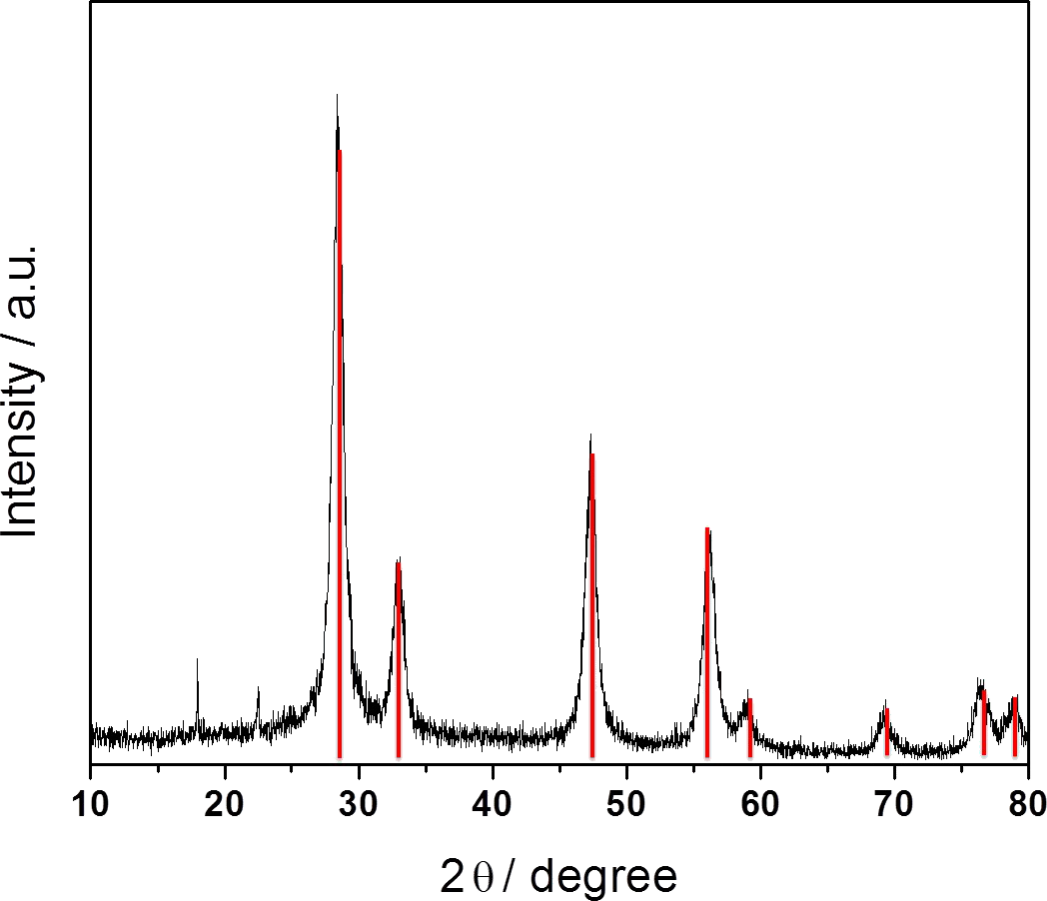
XRD pattern of hybrid polystyrene nanocapsules with cerium(IV) oxide on the surface (NC-CeO_2_). Vertical lines indicates the position and relative intensity of cubic cerium(IV) oxide crystal phase (ICDD card no. 34-0394).

**Figure 4:**
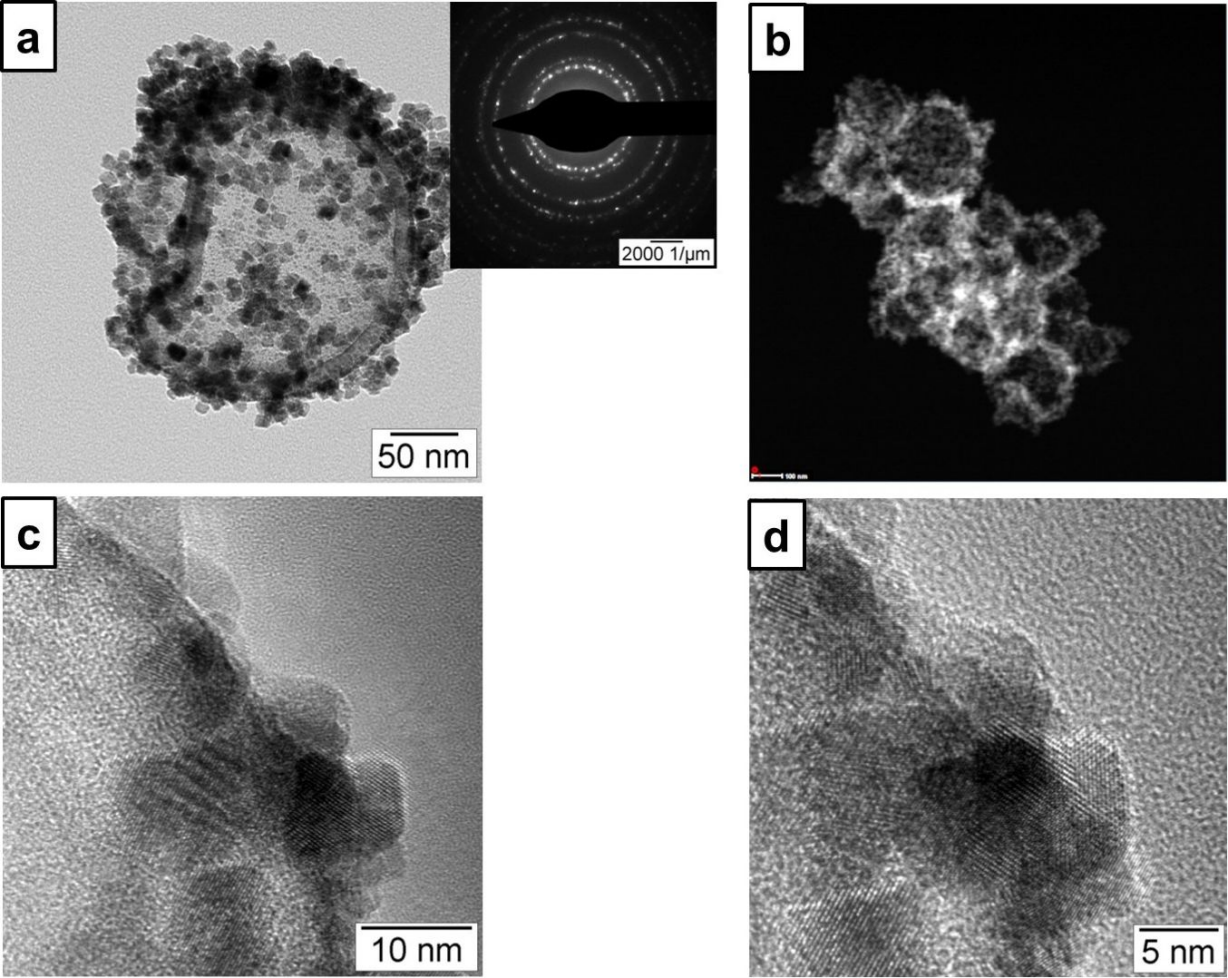
TEM micrographs of CeO_2_/polystyrene hybrid nanocapsules (sample NC-CeO_2_): a) bright-field image (inset shows the electron diffraction of the shown capsule); b) dark-field image; c, d) high-resolution images at different magnification.

The crystallization of cerium(IV) oxide nanoparticles on the surface of the polymer nanocapsules is depicted in Figure 1. First, the cerium ions from the precursor are complexed by the carboxylate functional groups and the crystallization occurs upon addition of NaOH at a controlled rate. The SEM and TEM images presented in Figure 2 demonstrate that the inorganic ceria nanoparticles were efficiently crystallized on the surface (Figure 2a,b shows the pristine sample NC and Figure 2c,d shows the hybrid sample, labeled as NC-CeO_2_). The X-ray diffraction (XRD) pattern of the hybrid sample, shown in Figure 3, was unambiguously assigned to crystalline CeO_2_ (ceria, JPCD card No. 34-0394). Elemental mapping by EDX also confirmed the presence of cerium in the investigated areas (see Supporting Information File 1, Figure S3). TEM images of ceria hybrid nanocapsules in Figure 4 indicate a homogeneous distribution of ceria nanocrystals on the surface of the capsules. The presence of bright spots in the dark field images (Figure 4b) confirms crystalline domains lying in the detection plane [10]. A corresponding high-resolution image of the hybrid sample NC-CeO_2_ is shown in Figure 4c,d. The ceria content for this sample was determined to be about 44 wt % by thermogravimetric analysis (see Supporting Information File 1, Figure S4).

**Figure 5:**
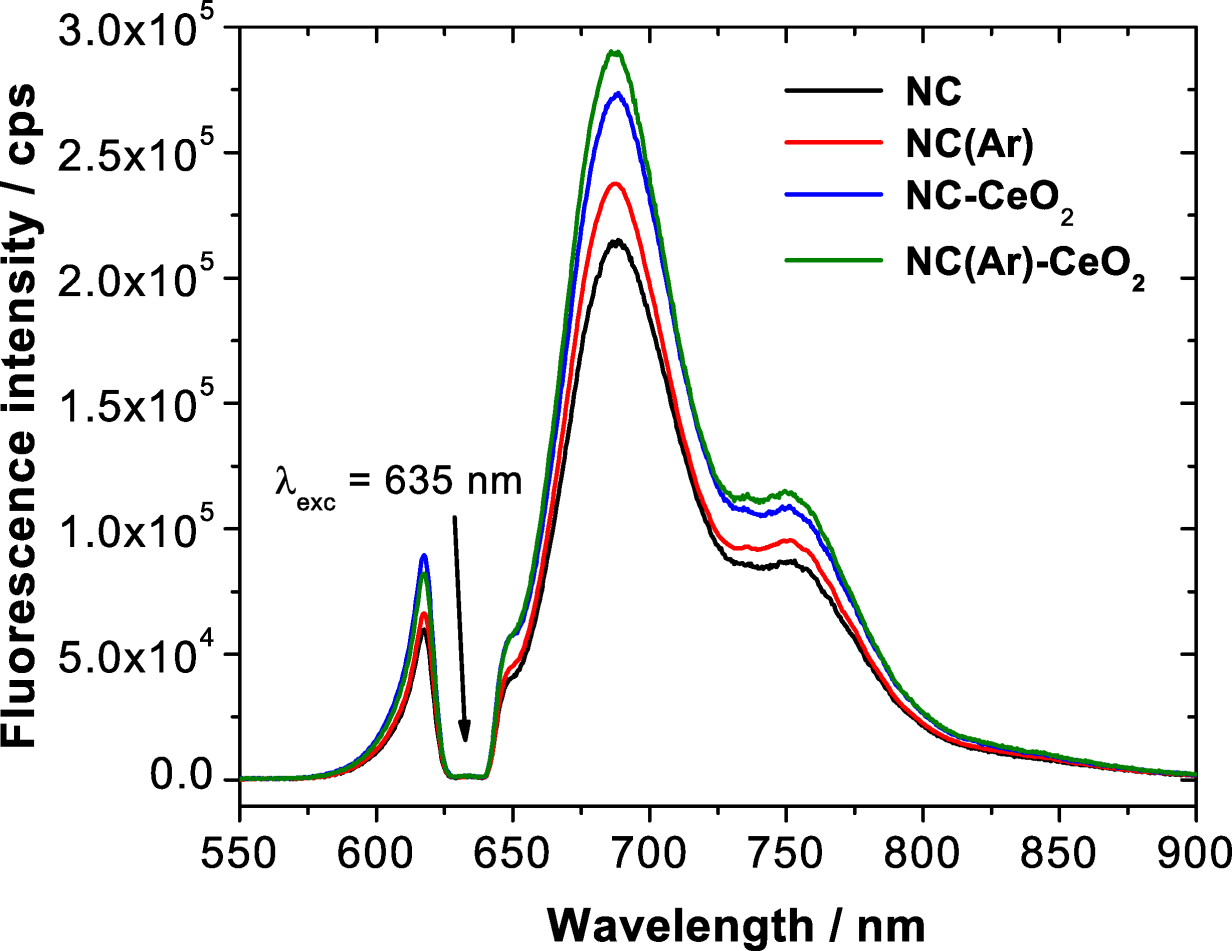
Fluorescence spectra of terylene diimide encapsulated in nanocapsules: NC (continuous black line, ambient conditions without cerium oxide), NC(Ar) (dashed red line, argon conditions without cerium oxide), NC-CeO_2_ (dotted blue line, ambient conditions with CeO_2_) and NC(Ar)-CeO_2_ (dash-dotted green line, argon conditions with CeO_2_).

The fluorescence spectrum measured under ambient conditions for the pure polymer nanocapsules (sample NC) is shown in Figure 5 (black line). An intense fluorescence emission from TDI with λ_max_ = 685 nm is observed. An important factor influencing the decrease of fluorescence efficiency is photooxidation. This kind of photochemical reaction is observed as a result of the interaction of the sample with singlet oxygen, which converts a fluorescent molecule into a state in which no longer absorbs and fluoresces. A possible explanation for the process of photooxidation in aromatic hydrocarbons is the non-zero probability for intersystem crossing. The excited triplet state is relatively long-lived, even at room temperature, and can serve as a source for generation of singlet oxygen.

In our case, during the encapsulation of TDI molecules the molecular oxygen present in the external environment (e.g., in the water phase) can enter into already formed polymer nanocapsules due to the permeability of the thin polymer shell. Thus, singlet oxygen is created by the process of interaction between electronic excited state of TDI and ground-state molecular oxygen within the encapsulated materials, which results in photo-oxidation processes. To overcome the limitations caused by oxygen influence, a sample named as NC(Ar) was prepared in a closed flask in argon atmosphere. The sample from the closed flask was transferred to a quartz cuvette under ambient conditions for further fluorescence measurements. As observed in Figure 5, the fluorescence efficiency of this sample is enhanced when compared to the sample prepared under ambient conditions, which is explained by the decrease of the oxygen concentration inside the capsules during polymerization. This decrease of oxygen content resulted also in a decreased rate of photodegradation of the TDI molecule.

To further minimize the effect of fluorescence quenching, cerium(IV) oxide was crystallized on the surface of polymer sample NC, which yielded the hybrid sample NC-CeO_2_. The fluorescence intensity is higher than for nanocapsules synthesized under ambient conditions without CeO_2_, but also even higher than for the sample NC(Ar) prepared under argon atmosphere. The oxygen vacancies present in the structure of cerium(IV) oxide nanoparticles are the most likely origin of the enhancement of the fluorescence, since they can scavenge oxygen molecules from the environment and prohibit molecular oxygen to enter into the nanocapsules.

Following these results, the deposition of cerium(IV) oxide was also carried out on nanocapsule samples prepared in inert atmosphere. The resulting sample was labeled as NC(Ar)-CeO_2_. The corresponding fluorescence spectrum is shown by the green curve in Figure 5. Control experiments were carried out to confirm that the improvement of fluorescence efficiency originates from the formed cerium oxide and not from the cerium(III) precursor itself or any of the precipitating agents (see Supporting Information File 1, Figure S5). An enhancement of the fluorescence intensity took place only in the presence of the crystallized cerium(IV) oxide.

To investigate the effect of external oxygen, we recorded again the fluorescence emission spectra for all synthesized samples after having sealed the samples with argon in the glove box (see Supporting Information File 1, Figure S6). The spectra did not show any significant difference to samples prepared in an oxygen-free atmosphere.

**Figure 6:**
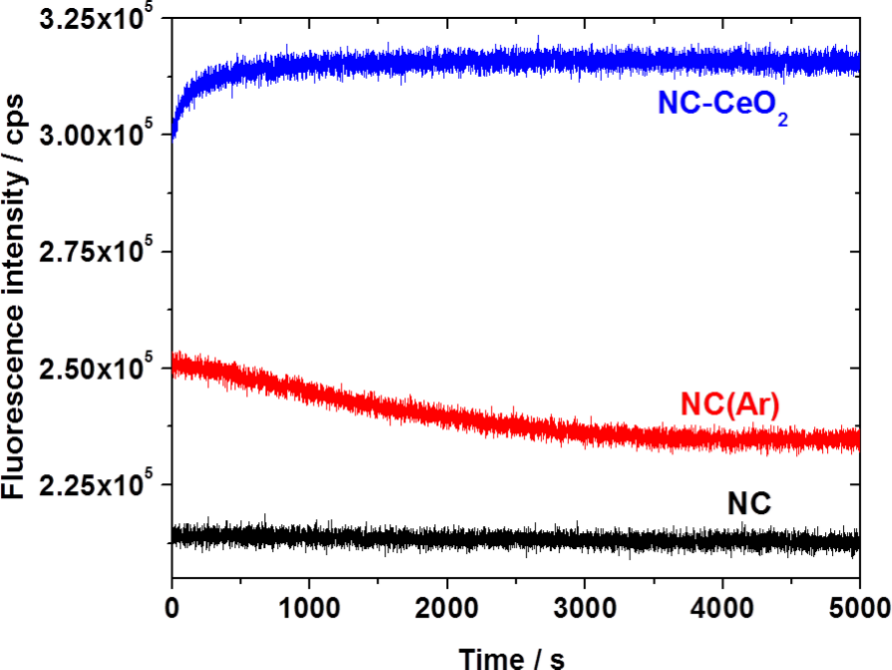
Photodegradation measurements of nanocapsules synthesized under different conditions without cerium oxide (sample NC at ambient conditions and sample NC(Ar) under argon) and with cerium oxide on the surface (sample NC-CeO_2_ at ambient conditions).

Finally, photodegradation experiments were carried out in an oxygen-rich environment to investigate the stability of the fluorescence emission of pure polymer nanocapsules synthesized under ambient conditions (NC) and in argon atmosphere NC(Ar) in comparison to the analogous hybrid nanocapsules with cerium(IV) oxide crystallized on the surface (NC-CeO_2_). The recorded spectra are shown in Figure 6. The fluorescence emission of NC(Ar) is less stable than that of NC-CeO_2_. Simultaneously, the fluorescence of sample NC-CeO_2_ is significantly more intense than the emission of the unprotected sample NC. After a sufficient time of excitation, sample NC(Ar) reaches the emission level of NC, while the CeO_2_ protection layer prevents effectively the oxygen penetration, leading to stable and efficient fluorescence even under ambient conditions. All these experimental observations point out that the presence of oxygen inside the nanocapsules is the most likely reason for the reduced fluorescence stability and intensity.

## Conclusion

In this work, we demonstrate that the armoring with CeO_2_ of polystyrene nanocapsules containing a model fluorophore molecule results in a significant enhancement of the fluorescence. The in situ crystallization of the metal oxide on the surface of the nanocontainers suppresses the photobleaching of the fluorescent molecule by molecular oxygen. Hybrid CeO_2_/polymer samples simply prepared under ambient conditions exhibit an even fluorescence intensity than pure polymer nanocapsules carefully prepared in an oxygen-free atmosphere. The enhancement effect can be explained by the trapping of the quenching oxygen molecules on the metal oxide surface, which results in a reduction of the photo-oxidation.

## Supporting Information

File 1 Chemical structure of TDI, absorption and emission spectra of TDI, EDX spectra, TGA, and additional photoluminescence emission spectra of samples.
