# Supplementary material for: Ceria/polymer nanocontainers for high-performance encapsulation of fluorophores
Source: Beilstein J Nanotechnol. 2019 Feb 22;10:522–30. doi: 10.3762/bjnano.10.53 (PMC6404412; doi:10.3762/bjnano.10.53)
Supplement: File 1 — Chemical structure of TDI, absorption and emission spectra of TDI, EDX spectra, TGA, and additional photoluminescence emission spectra of samples. [file Beilstein_J_Nanotechnol-10-522-s001.pdf]

BEILSTEIN JOURNAL OF NANOTECHNOLOGY

Supporting Information

for

Ceria/polymer nanocontainers for high-performance encapsulation of fluorophores

Kartheek Katta, Dmitry Busko, Yuri Avlasevich, Katharina Landfester, Stanislav Baluschev and Rafael Muñoz-Espí

Beilstein J. Nanotechnol. **2019,** *10,* 522–530. doi:10.3762/bjnano.10.53

Chemical structure of TDI, absorption and emission spectra of TDI, EDX spectra, TGA, and additional photoluminescence emission spectra of samples

License and Terms: This is a supporting information file under the terms of the Creative Commons Attribution License (http://creativecommons.org/licenses/by/4.0). Please note that the reuse, redistribution and reproduction in particular requires that the authors and source are credited.
The license is subject to the *Beilstein Journal of Nanotechnology* terms and conditions: (https://www.beilstein-journals.org/bjnano)

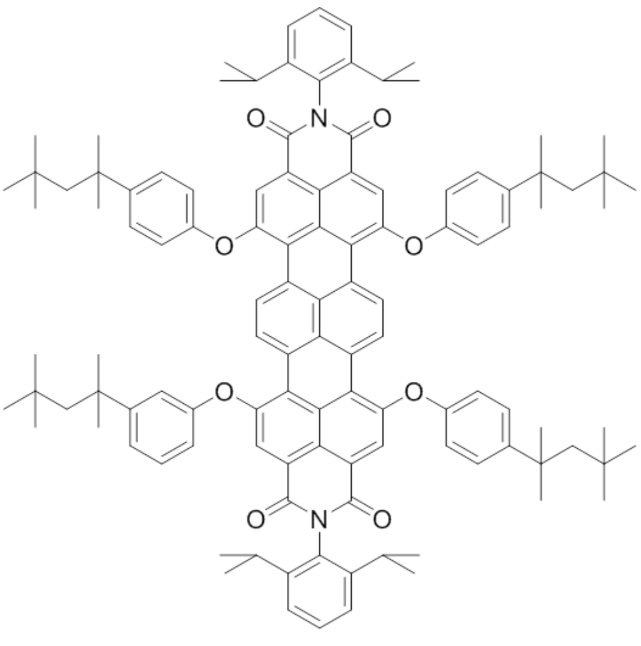

Figure S1: Chemical structure of *N,N*'-(2,6-diisopropylphenyl)-1,6,9,13-tetrakis[4-(1,1,3,3-tetramethylbutyl)phenoxy]terrylene-3,4,11,12-bis(dicarboximide) (TDI)

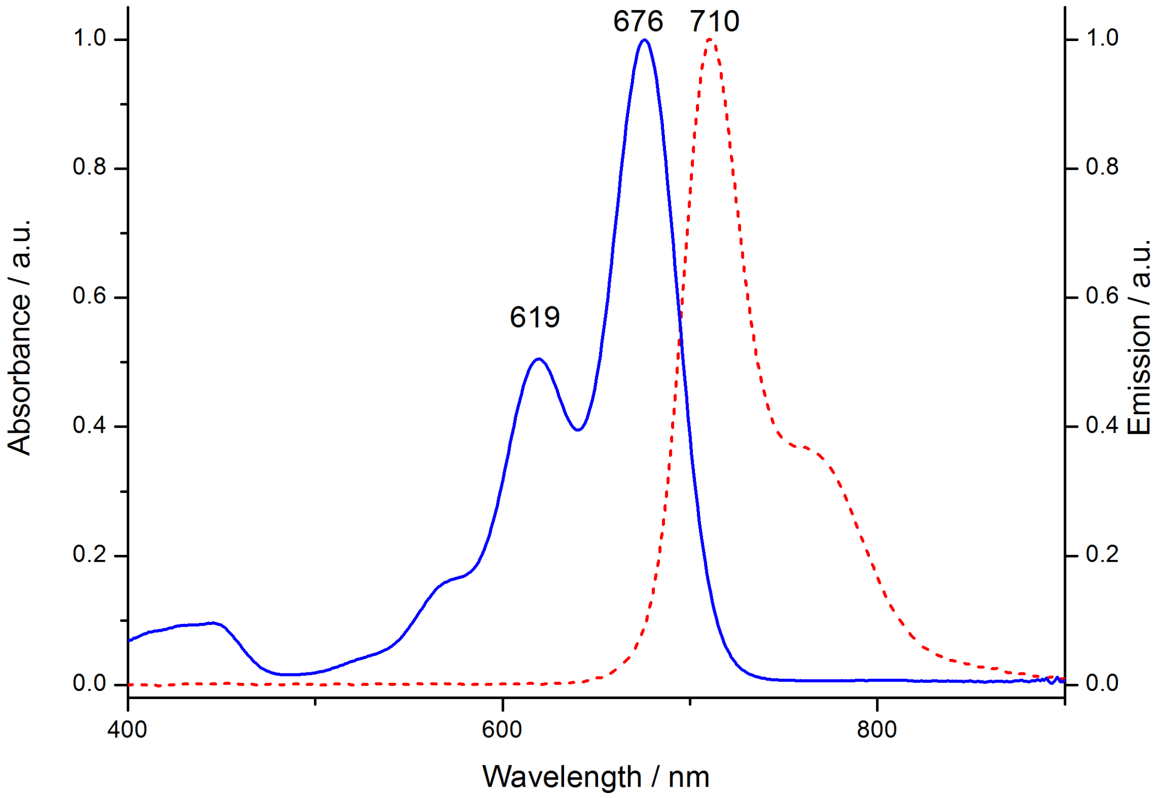

Figure S2: Absorption (blue curve) and fluorescence emission (red curve) spectra for TDI.

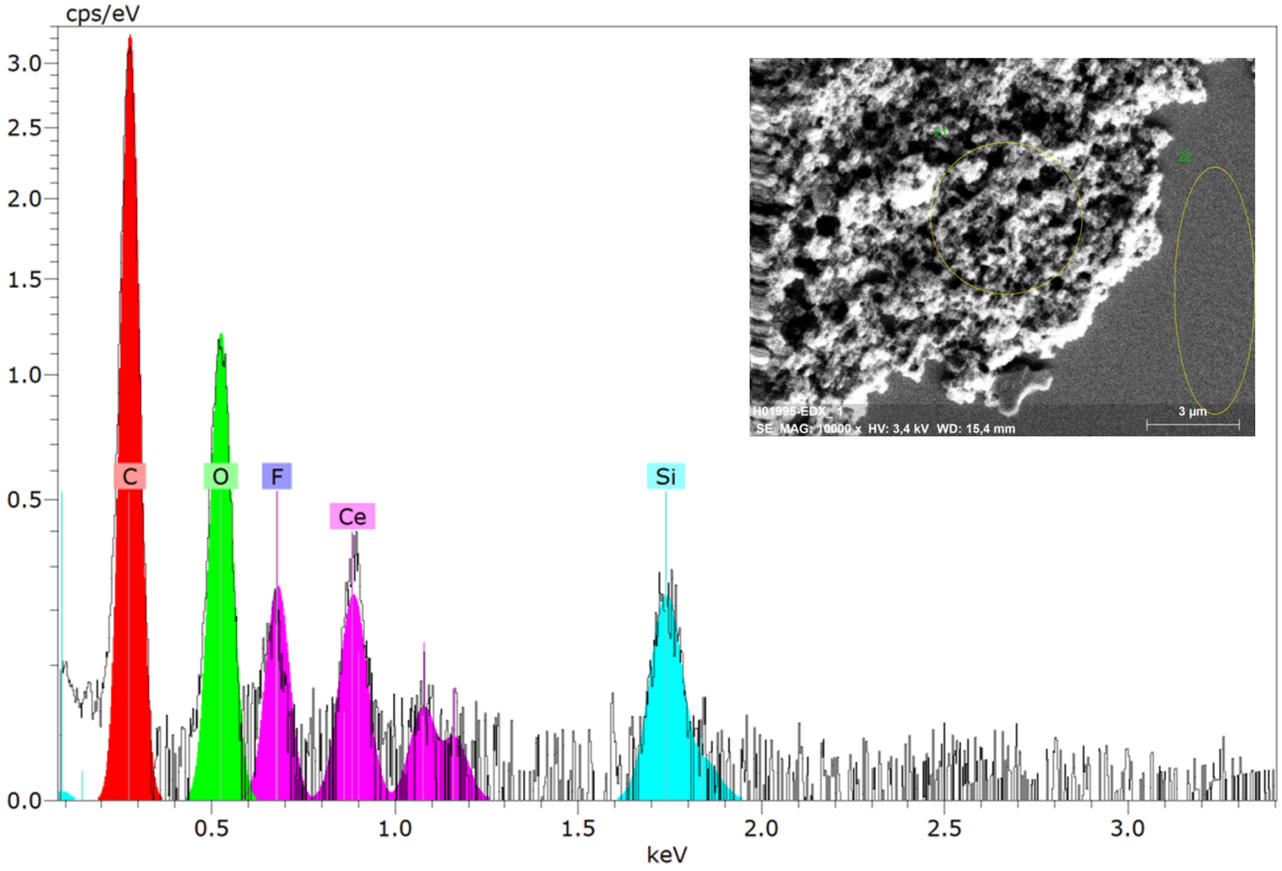

Figure S3: EDX spectrum and corresponding SEM image of hybrid polystyrene nanocapsules with CeO_2 on the surface (sample **NC-CeO_2**).

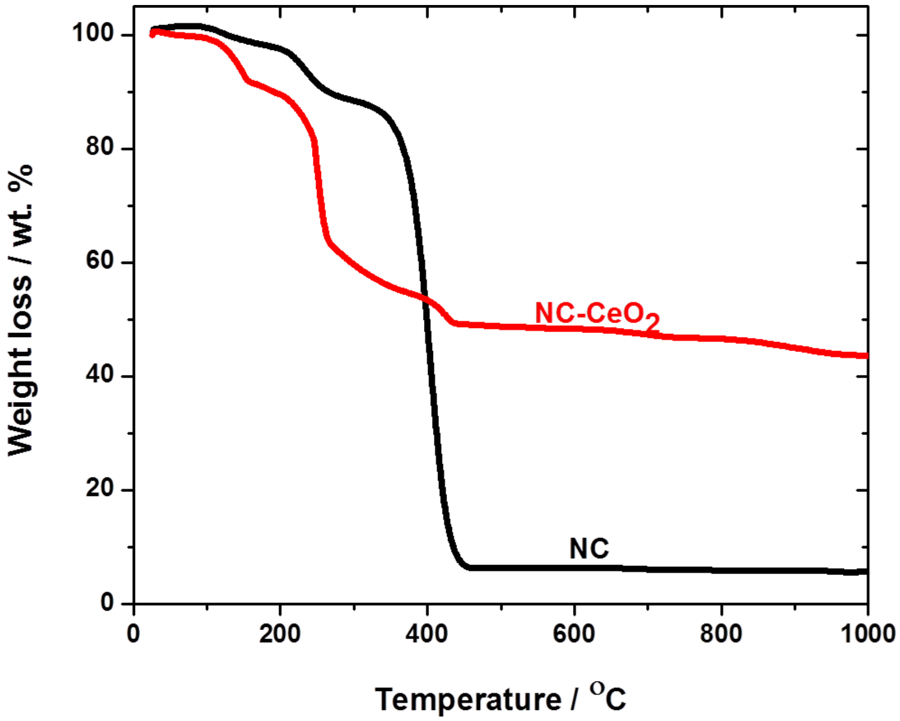

Figure S4: Thermogravimetric analysis curves of **NC** (black line, ambient conditions without cerium oxide) and **NC-CeO$_2$** (red line, ambient conditions with cerium oxide).

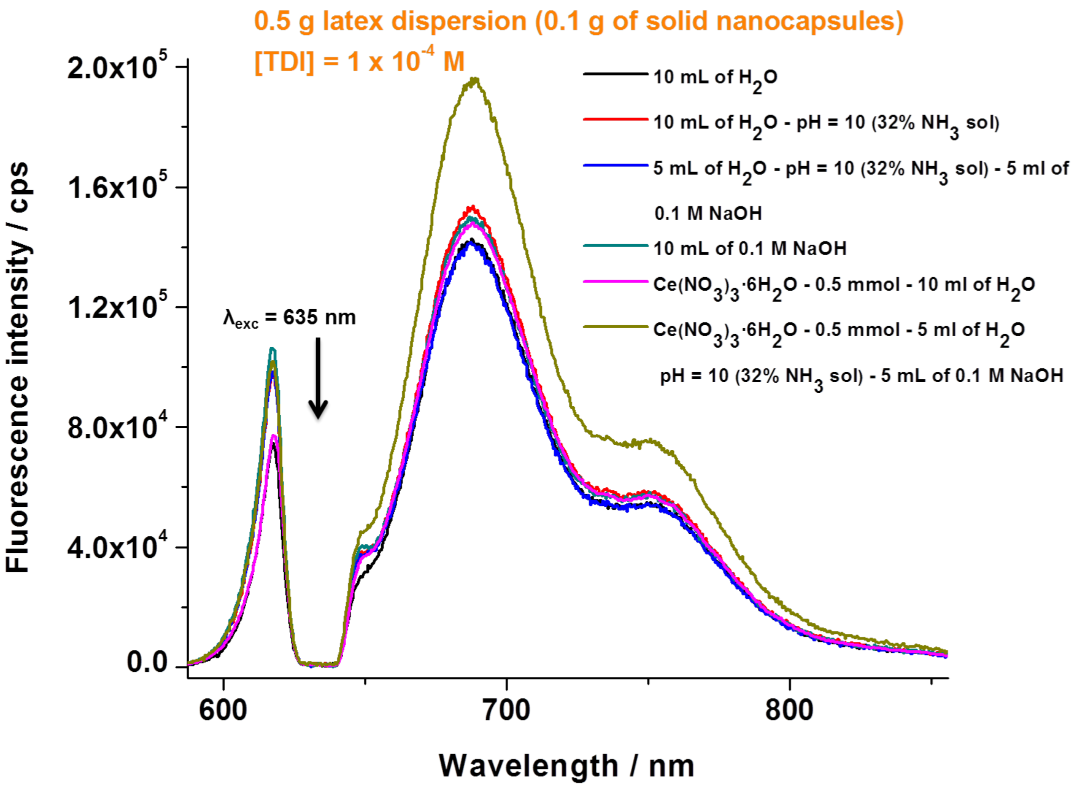

Figure S5: Luminescence spectra of terylene diimide encapsulated nanocapsules under various conditions.The excitation intensity of the laser (635 nm) was 0.2 W cm^{-2}.

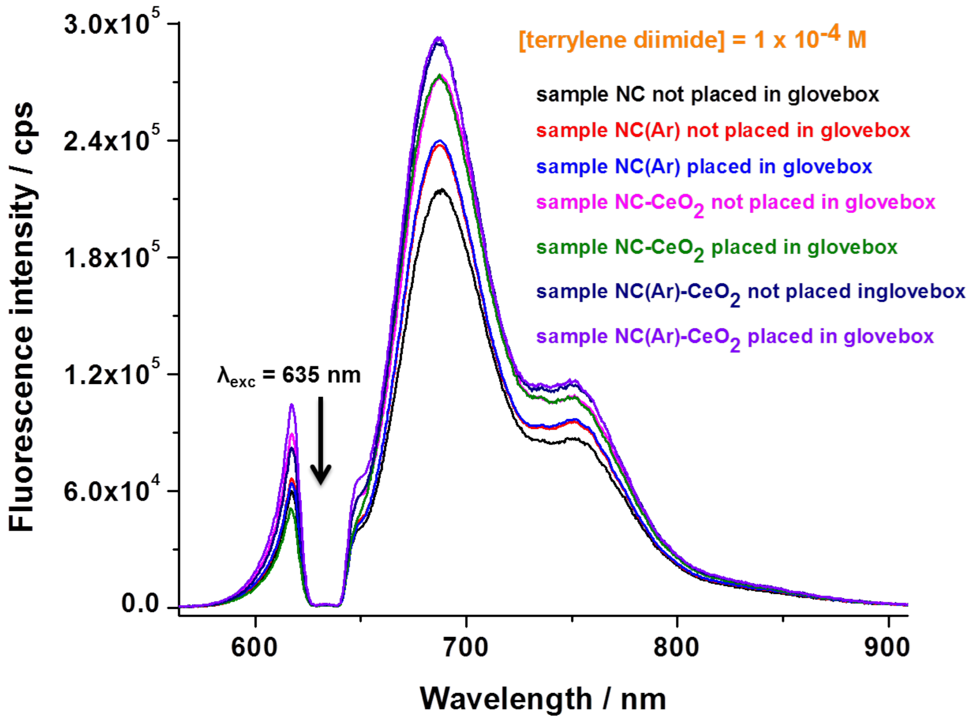

Figure S6: Luminescence emission spectra of samples encapsulating lated terrylene diimide, placed and not placed in glovebox: **NC** (ambient conditions without CeO_2), **NC(Ar)** (argon conditions without CeO_2), NC-CeO2 (ambient conditions with CeO_2), and **NC(Ar)-CeO$_2$** (argon conditions with CeO_2). The excitation intensity of the laser (635 nm) was 0.2 W cm^{-2}.
